# What we may learn – and need – from pandemic fiction

**DOI:** 10.1186/s13010-020-00089-0

**Published:** 2020-07-21

**Authors:** Jane Doherty, James Giordano

**Affiliations:** Departments of Neurology and Biochemistry, and Pellegrino Center for Clinical Bioethics, Georgetown University Medical Center, Washington DC, USA

**Keywords:** Science fiction, Pandemic, Cognition, COVID-19, SARS-CoV2

## Abstract

N/A

During this pandemic, many have found themselves glued to the news searching for a better understanding of the current situation. People are desperate for factual information to either assuage or verify their fears. This sentiment is exemplified in the very nomenclature of the abundant programs covering COVID-19 [1]. But this begs the questions: does fact always trump fiction? And, are they actually at odds with one another?

First, it’s important to understand that fiction is not the same as a lie [2, 3]. To be sure, facts trump lies. While both deal with the unreal, a lie is intentionally opposed to some truth, whereas fiction intends to provide an illustration – albeit through implicit meanings and conclusions – of reality. Since the global outbreak of COVID-19, internet users’ interest in movies about pandemics has increased by 4900% [4]. The timing and the magnitude of this increased interest makes clear that science fiction stories offer more than simply entertainment. Instead, people may be engaging with movies like *Contagion* [5], *Outbreak* [6], *The Andromeda Strain* [7]*, Flu* [8]*,* and *Virus* [9] in ways similar to those reasons for which they turn to the news: a desire and search for deeper understanding and some sense of security from things unknown.

Literary enthusiast Patrick Parrinder describes science fiction as a “thinking machine” that provides an outlet to visualize *what could be,* and therefore allows both reflection on *what is,* and some idea for planning *what to do next* [10]*.* This sheds some light upon the current interest in pandemic fiction. These movies engender deeper contemplation of the COVID-19 crisis because they combine the familiar with the novel. Balancing the two - both the factual and fictitious - is critical to instilling a sense of *ostranenie* [11]: the unfamiliar presentation of a common thing that affords the viewer an enhanced perception of the familiar. Science fiction stitches truths about humanity into the fabric of its unfamiliar worlds: when we imagine ourselves in stories’ novel scenarios, it provides good food for thought and the possibility to internalize applicable moral lessons.

As such, the pandemic movies currently claiming top spots on streaming services allow us to ponder some pertinent questions:

## How does fear influence us?

Fear is central to any portrayal of a pandemic. These movies allow viewers to more carefully examine the basis, uses, and pitfalls of fear. For example, in the film *Outbreak* [6], Dustin Hoffman’s character states that “fear gets a bad rap.” When things are truly scary, fear serves as a reminder of human vulnerability. In this way, fear is akin to a litmus test, indicating what we truly care about, dread losing, and want to protect. But fears are not always based in reality, and such misapprehension can be counterproductive. The films *Contagion* [5] and *Flu* [8] get audiences to question the source of fear, and recognize the danger of misinformation. This is accomplished through dialogue such as “… in order to become sick, you first need to come in contact with the disease. In order to get scared, all you have to do is come in contact with a rumor … spreading [rumors] is far more dangerous than the disease” [5]. Fiction enables us to examine our fears and sources of information about COVID-19.

## Who do we turn to for answers?

A consistent plot line in almost all pandemic fiction involves a struggle for authority between a scientist and a politician. Pandemic movies depict the pendulum of peoples’ trust in scientists: swinging from a belief that scientists are “Jesus in a lab coat”, to a complete lack of confidence, if not outright disdain [5]. Ultimately, through plot resolution, these conflicting views are smoothed. Pandemic movies teach that these problems cannot be solved without science, and in so doing compel us to strengthen appropriately balanced collaboration between scientists and other authority figures.

## In a world where many are suffering, who gets treatment?

With mass infection, these fictitious worlds’ resources are stretched thin, raising the ethical dilemma of how to distribute the limited aid available. As recent headlines have revealed, life and art are not so distinct. Pandemic movies expose the impact that our urge to protect loved ones can have on the equity of resource distribution. This turns the lens of film into a mirror upon our own values, and the ways that we regard self, kin, kith, and others. The characters in *Outbreak* [6]*, Contagion* [5]*,* and *Flu* [8] are compelled to treat either themselves, their families, or “their own people” first. But as so well expressed in *Contagion*, “… we all have people” [5]. These storylines encourage us to question who we consider in- and/or out-groups, and prompt us to query such distinctions in order to ethically care for and treat humanity at-large.

The “thinking machine” that is pandemic science fiction evokes intentional reflection on these pertinent questions. But what if these fictional portrayals of pandemics are doing more to help comprehend this crisis and how we think and behave in response? Neuroscientific investigations of imagination and visualization reveal how fiction might affect thought processes in more subtle ways [12–17]. Fiction, long cherished for its ability to transport its audience to different worlds and into the minds of characters [18], is highly dependent upon the brain’s capacity for visualization. Visualization is the mental exercise of imagining what is not depicted in our local reality [19]. Several studies have linked the practice of visualizing to cognitive functions beyond the mere ability to enjoy a story. They demonstrate that the act of mental simulation or visualization increases the likelihood of believing the visualized events will occur in the future [14], can aid emotional regulation and emotional coping, and improve problem-solving and planning capabilities [12].

A Harvard University study [14] showed that thinking about an emotion engages the same neurological networks as talking about and feeling one’s actual emotions. For sure, COVID-19 has imposed new emotional strains [20]. Thus, it is possible that mentally stimulating or viewing these emotions – via film - could be a form of coping that leads to greater emotional regulation. So, it seems that viruses aren’t the only contagious thing being depicted in pandemic movies: emotions can be “transferred” from the screen to our brains just as quickly as the fictional disease spreads from one character to another. When we watch pandemic fiction, we are mentally rehearsing the range of emotions being depicted in these films. Common throughout all are feelings of fear, confusion, desperation, and isolation – a simulacrum of those currently so prevalent. By dealing with these emotions through fiction, the imagination involved may help us process our intense feelings and afford capabilities to better navigate these tumultuous times.

A UCLA study, *Harnessing the Imagination*, revealed that problem-solving skills improved when participants used specific visualization techniques [12]. To be effective, visualization had to involve mentally engaging in steps that individual participants thought were necessary to accomplish their goals. Simply picturing some magical solution to their problem was ineffective. This need for realism is further supported by the work of Daniel Kahneman and Dave Miller who observed that to be productive, visualization ought “not to rely on improbable or fantastic steps along the way” [21].

Applying this outcome of visualization - improved problem-solving and planning skills - to the experience of mentally processing a science fiction storyline can get a little murky. Could making pandemic movies follow more realistic, scientifically-informed plots improve our ability to plan for the future? Further research is needed to examine whether watching the problem-solving process portrayed in a movie provides equivalent benefits to mentally rehearsing the goal-productive steps as demonstrated in the UCLA study [12] Perhaps the impetus for such studies lies in scientifically exploring the validity of the sentiment that “you become what you think about all day long” [22].

Of course, science fiction is allowed, if not required, to break the rules of what is true, and wander into the realm of the unreal. Let’s not forget that the unfamiliar is what makes fiction so powerful and appealing. While acknowledging that science fiction is entitled to breaches in reality, we should keep in mind the intricate interdependence of fiction and fact. When science fiction is used as a tool to grapple with current realities - as exemplified by the resurgent popularity of pandemic movies - the genre takes on an additional obligation: to honor, and positively leverage, the reciprocity between works of fiction and our perception of fact. By creating stories that are rooted in scientific fact and that retain cultural relevance, we can maximize science fiction’s positive impact on our real-world response to this pandemic. In this way, fact-informed fiction can lead us to engage in more meaningful reflection of stories’ object lessons and through them, build a better fiction-informed future.

## Data Availability

N/A
